# Investigating the shift between externally and internally oriented cognition: a novel task-switching paradigm

**DOI:** 10.1093/nc/niac016

**Published:** 2022-11-19

**Authors:** Sara Calzolari, Svetla Boneva, Davinia Fernández-Espejo

**Affiliations:** Centre for Human Brain Health, University of Birmingham, 05 CHBH Building, Edgbaston, Birmingham B15 2TT, UK; Centre for Human Brain Health, University of Birmingham, 05 CHBH Building, Edgbaston, Birmingham B15 2TT, UK; School of Psychology, University of Birmingham, 05 CHBH Building, Edgbaston, Birmingham B15 2TT, UK; Centre for Human Brain Health, University of Birmingham, 05 CHBH Building, Edgbaston, Birmingham B15 2TT, UK; School of Psychology, University of Birmingham, 05 CHBH Building, Edgbaston, Birmingham B15 2TT, UK

**Keywords:** self-oriented cognition, task switching

## Abstract

Despite our constant need to flexibly balance internal and external information, research on cognitive flexibility has focused solely on shifts between externally oriented tasks. In contrast, switches across internally oriented processes (and self-referential cognition specifically) and between internal and external domains have never been investigated. Here, we report a novel task-switching paradigm developed to explore the behavioural signatures associated with cognitive flexibility when self-referential processes, as well as more traditional external processes, are involved. Two hundred healthy volunteers completed an online task. In each trial, participants performed one of four possible tasks on written words, as instructed by a pre-stimulus cue. These included two externally and two internally oriented tasks: assessing whether the third letter was a consonant or the penultimate letter was a vowel versus assessing whether the adjective applied to their personality or if it described a bodily sensation they were currently experiencing. In total, 40% of trials involved switches to another task, and these were equally distributed across within-external, within-internal, internal-to-external and external-to-internal switches. We found higher response times for switches compared to repetitions both in the external and internal domains, thus demonstrating the presence of switch costs in self-referential tasks for the first time. We also found higher response times for between-domain switches compared to switches within each domain. We propose that these effects originate from the goal-directed engagement of different domain-specific cognitive systems that flexibly communicate and share domain-general control features.

## Introduction

Human beings deal with a continuous flow of sensory stimuli coming from our environment, as well as interoceptive sensations and spontaneous thoughts. Our brain is thus constantly integrating and coordinating external and internal inputs in a dynamic manner. The field of cognitive psychology has widely used task-switching paradigms to investigate our ability to flexibly shift between different cognitive and attentional processes (Kim *et al.* 2012). In these paradigms, two or more tasks are performed sequentially, either with a predictable sequence or in random alternation. Participants typically show worse performance in trials with different task demands to the previous trial (switch) compared to trials preceded by the same type of task (repetition) – a phenomenon termed ‘switch cost’ (Koch *et al.* 2018). Switch costs represent the cognitive costs associated with task-set changes in a context where other active competing task sets are interfering (Dove *et al.* 2000) and usually translate into higher response times (RTs) and/or error rates in switch trials compared to repetitions.

Despite the shift between external and internal worlds being a ubiquitous and fundamental feature of our brain and cognition, research on cognitive flexibility to date has almost exclusively focused on switches between externally oriented tasks (e.g. categorizing digits based on parity or magnitude and stimuli based on colour or shape; Koch 2003; Schneider and Logan 2010). An exception to this is a series of recent studies by Verschooren *et al.* that aimed to understand how the attentional switches between traditionally used external tasks (in this case perceptual attention) and those requiring working memory processes (i.e. when no external stimulus is present during the task) occur (Verschooren *et al.* 2019a,b). They found that costs are also present when switching between these two types of tasks, although of similar magnitude compared to switches within the perceptual domain (Verschooren *et al.* 2019b), and that such costs are asymmetric (i.e. greater when switching towards their working memory task compared to switching to the perceptual attention task; Verschooren *et al.* 2019a). While Verschooren’s work elegantly demonstrates the existence of costs between domains, their internal attention/working memory task required the internal manipulation of previously presented digits [see also Gilbert *et al.* (2005) for a similar line of research]. Instead, we here focus on self-referential cognition, encompassing physical and psychological aspects such as representation and awareness of one’s own body and bodily actions, autobiographical and semantic memory, personal traits and subjective perspective (Gillihan and Farah 2005; Christoff *et al.* 2011; D’Argembeau 2020). There is vast literature investigating self-referential processes (Gillihan and Farah 2005; Northoff *et al.* 2006; Knyazev 2013) but, to our knowledge, the switch between different types of self-referential tasks or between these tasks and externally oriented tasks is yet to be explored. Neuroimaging research has demonstrated that internal and external processes are subserved by different brain networks (Northoff and Bermpohl 2004; Benoit *et al.* 2010; Mäki-Marttunen *et al.* 2016). The relationship between these networks is characterized by complex dynamics (Kelley *et al.* 2002; Weissman *et al.* 2006; Fox and Raichle 2007; Vanhaudenhuyse *et al.* 2010; Whitfield-Gabrieli *et al.* 2011; Davey *et al.* 2016), suggesting that switching across the two domains might be more costly than the traditionally reported switches between external tasks.

The study of self-reference often entails the comparison between stimuli that participants recognize as referring to themselves and stimuli attributed to others or simply not recognized as one’s own (Kawahara and Yamada 2004; Brédart *et al.* 2006; Devue and Brédart 2008). A common experimental paradigm requires participants to judge whether a series of traits apply to them or not. This is usually accompanied by control conditions such as judging if the trait applies to someone else (e.g. a famous public figure or a friend) and/or focusing on purely linguistic or stylistic attributes of the words such as counting letters or discriminating case or font (Kelley *et al.* 2002; Davey *et al.* 2016; Liu *et al.* 2017).

Here, we report a novel task-switching paradigm that requires either attention towards external stimulus features or an internal, self-related focus.

Based on the above-mentioned literature and our own (unpublished) pilot data, we hypothesized the following:

Hypothesis 1: We will find evidence of an overall switch cost: i.e. switching from one task to another will result in higher RTs compared to repeating the same task, irrespective of task type.

 

Hypothesis 2: Internally oriented tasks will also exhibit switch costs: i.e. switching between self-referential tasks will result in higher RTs as compared to repetitions.

 

Hypothesis 3: Switch costs involving self-referential tasks will be similar to those involving externally oriented tasks.

 

Hypothesis 4: Switching between similar tasks (i.e. within the internal or external domain) will be associated with faster RTs than switching between more distant cognitive processes or domains, i.e. from externally to internally oriented tasks, and vice versa.

Finally, we explored whether the difference in within- versus between-domain switches changes as a function of the direction of the switch (i.e. when the switch goes towards an internal task versus an external task).

## Materials and methods

We pre-registered our methods, research hypotheses and planned analyses prior to data collection on the Open Science Framework (OSF) (https://osf.io/p7xrq). Any deviations from the pre-registration are noted.

### Participants

We recruited a total of 230 participants (mean age = 28.29 ± 4.5; 144 women, 85 men and 1 non-binary). To be eligible, participants had to be aged 18–35 years, right-handed, native English speakers, with no history of neurological or psychiatric conditions nor sleep problems (insomnia/hypersomnia), and a normal sleep schedule (e.g. no night-shift jobs). Participants enrolled in the study via Prolific (www.prolific.co) and received compensation upon completion of the study at a rate of £5 h per hour. They received written information and provided written informed consent before participating. This study was approved by the University of Birmingham’s Science, Technology, Engineering and Mathematics ethics committee.

We discarded 5 participants based on not actually meeting eligibility criteria (4 were not monolingual British English speakers and 1 had a diagnosis of dyslexia), 1 for having >10% of missing trials and 24 due to low accuracy (<60%) in one or more task conditions. This resulted in a total of 200 participants (mean age = 28.49 ± 4.4; 126 women, 73 men and 1 non-binary) included in the analysis.

As per our pre-registration, after obtaining 200 valid datasets, we applied a Bayesian stopping rule (see the Statistical Analysis section) to decide whether to continue data collection. Specifically, a Bayes factor (BF_10_) of ≥3 (either in support of the alternative or the null hypothesis) in the *t*-test comparing RTs during repetition trials versus all types of switch trials (i.e. evidence of overall switch costs, *Hypothesis 1*) would mean that data collection could be stopped. As we obtained very strong evidence in support of the presence of a switch cost (see the Results section), we did not require any further participants.

### Experimental design and procedure

**Figure 1.**
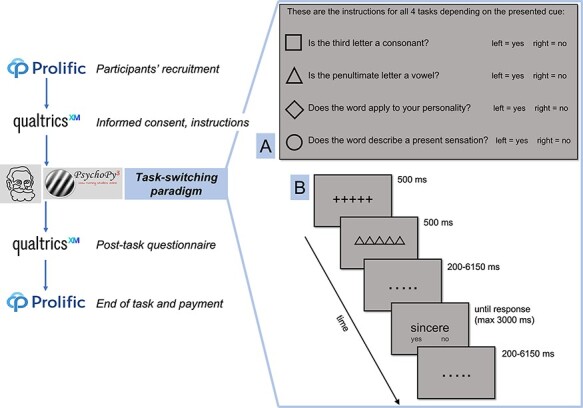
Online study pipeline (left) and details of the main paradigm (right) including an instruction screen showing one of the four possible cue-task mappings (**A**) and the structure of a single trial (**B**)

We adopted a within-subjects design. Participants completed all conditions of the behavioural experiment online in one session lasting ∼1 h and 20 min (Fig. 1) and could only participate using a computer or laptop (no phones or tablets). From Prolific, participants directly accessed an online form on Qualtrics (Qualtrics, Provo, UT, USA), where they received written information regarding the study, gave informed consent, provided demographic data and carefully read the task instructions. They were then automatically redirected to Pavlovia (www.pavlovia.org), where they performed an initial training session and the task-switching paradigm for ∼1 h (10 min for training and 50 min for the main experiment, see the next subsection for details). After completion, they were redirected to Qualtrics for a final questionnaire aimed at verifying the correct responses for the two internal task conditions. This lasted ∼10 min. Specifically, participants were asked to perform the same two internal tasks again (‘personality’ and ‘current sensations’ tasks, as described in the next subsection) in a self-paced questionnaire that displayed all stimuli at once in a list. We used this as a proxy to calculate participants’ accuracy in these tasks on the main paradigm: i.e. we considered correct only those responses that matched the ones given on the questionnaire.

### Task

On each trial, participants had to complete one of four possible tasks on a written word presented on the screen, with a geometric cue appearing before each stimulus to indicate which task to perform. We designed the tasks to induce attention towards external stimuli (2 ‘external’ tasks) or towards self-related characteristics (2 ‘internal’ tasks). We used the same stimuli and response format throughout the paradigm: personality traits and bodily adjectives presented in written form on the screen that required binary yes/no responses. The four tasks were the following:

- Consonant finding (External Task 1): participants had to assess whether the third letter of the written form of the word was a consonant.
- Vowel finding (External Task 2): participants had to assess whether the penultimate (second to last) letter of the written form of the word was a vowel.
- Personality (Internal Task 1): participants had to report whether the adjective described their own personality, focusing on how their character is in everyday life.
- Current physical sensations (Internal Task 2): participants had to report whether the adjective described their present sensations, with a focus on current feelings from their body.

We designed our tasks to be as balanced as possible and not have intrinsic differences in difficulty (based on pilot data). Therefore, we expected the performance in task repetition trials to be similar across tasks.

The experiment included 381 trials in total (95 for each task except the consonant task which had 96) to ensure an even number of switches and repetitions, since the first trial did not belong to either category and was therefore not considered for the analysis. Trials were pseudo-randomly shuffled for each participant in order to obtain 60% repetition trials (i.e. trials where participants repeated the same task done in the previous trial) and 40% switch trials (i.e. trials where the task was different than the previous trial). Repetition trials were evenly distributed to obtain 50% of internal task repetitions and 50% of external task repetitions. Among the switch trials, one-fourth were switches between the two internal tasks, one-fourth between the two external tasks, one-fourth went from an external to an internal task and one-fourth from an internal to an external task. Participants had the chance to have a self-paced break every 95 trials (once every ∼12 min).

The initial training phase consisted of 60 trials divided in 5 blocks: the first four allowed participants to become familiar with each task separately and included 6 trials each; the last block included 36 switch trials with all four tasks.

All stimuli appeared in black at the centre of a grey screen. In each trial, a string of black fixation crosses (‘+++++’) first appeared for 500 ms, followed by a string of five black geometrical shapes (500 ms) that instructed participants about which task to perform (see Fig. 1). Specifically, cues were circles, squares, triangles or diamonds, and the assignment of each shape to a specific task instruction was randomized across participants, drawing from four possible cue-task pairing combinations. The presence of anticipatory cues allows for the engagement of preparatory processes in advance of the upcoming task, as compared to reactive processes only (Koch and Allport 2006). It also allowed us to use the identical stimuli across tasks. Target stimuli (words) appeared on the screen after a pseudorandom cue-to-target interval (CTI) and stayed on-screen until participants made a response, up to a maximum of 3 s. All trial elements were scaled based on the screen: fixation crosses maintained a height equal to 8% of screen size, stimuli of 4.5%, and cues were ∼5.5% of screen size (with slight variations to level off the different shapes). During the CTI and inter-trial interval (ITI), participants saw a grey screen with five black dots in the centre. CTI and ITI were pseudo-logarithmically jittered according to previous task-switching paradigms (De Baene and Brass 2011), with intervals ranging from 200 to 6150 ms in steps of 350 ms. Specifically, 50% of the trials ranged between 200 and 1950 ms, one-third of the trials between 2300–4050 ms, and the remaining one-sixth of trials between 4400 and 6150 ms. Since the time available for task preparation (i.e. the CTI) was shown to influence the costs of task-switching (Kiesel *et al.* 2010), we decided to employ such jittering method in this behavioural study as well to maintain consistency with future functional magnetic resonance imaging studies.

We prepared the stimuli lists, CTI and trial order using MATLAB (R2018b), coded the experiment using PsychoPy (Peirce *et al*. 2019)* * and delivered it to participants via Pavlovia (https://pavlovia.org/).

### Stimuli

Stimuli were all English adjectives pooled from Anderson’s list of 555 personality-trait words (Anderson 1968) and from a recently published list of concepts rated for interoceptive strength (Connell *et al.* 2018). We gathered a series of psycholinguistic variables and ratings for the selected words, which we used for the creation of well-balanced individual lists. These included the number of syllables, letters and phonemes from the Medical Research Council psycholinguistics database (Coltheart 1981) and the N-Watch software (Davis 2005); valence and arousal ratings from an extended version of the Affective Norms for English Words database (Warriner *et al.* 2013); interoception ratings from Connell *et al*. (2018); frequency ratings from the Subtitle-based word frequencies for British English database (Van Heuven *et al.* 2014)* *and mean age of acquisition ratings from Catling and Johnston (2009). We also labelled each word as congruent or incongruent depending on whether it was associated with the same yes/no response to the vowel and the consonant finding tasks (to control for possible congruency effects on RTs).

With a custom MATLAB script, we first randomly sorted the words into four lists (one per task). Then, we performed a 1 × 4 analysis of variance (ANOVA) on each of the above-mentioned variables to check that these four lists were not significantly different from each other in any of the above-mentioned variables. Interoception ratings were the only exception: by necessity, the lists for the current sensations task had higher interoceptive ratings than the personality task to encourage more embodied feelings in one task versus more abstract characteristics in the other. In addition to controlling for confounding variables, the lists assigned to the consonant finding task had approximately half of the words with a consonant in the third position, while the lists for the vowel finding task had approximately half of the words containing a vowel in the penultimate position (to ensure balanced responses throughout the experiment). In doing this, we allowed for a small difference of ±3 words to ensure that reaching a solution was computationally feasible. Importantly, the lists for the two external tasks each consisted of half trait and half bodily adjectives, while the personality and current sensations tasks were entirely composed of trait and bodily adjectives, respectively. This full procedure was iteratively repeated until 70 suitable lists were obtained. From these, the platform sequentially assigned a list for each participant. As there were more participants than available lists, some participants received the same list. However, as the lists contained unique words (i.e. no words were repeated within or across lists), for each participant each word only appeared once.

For the training phase trials (before the main task), we only controlled stimuli for the number of letters, frequency, valence and responses to external tasks, using the same procedure described earlier. In the case of valence, when ratings were missing, we used ratings from the most similar words available, e.g. ‘relieved’ from ‘relieve’. Note that this only applied to the training list, and for the main experiment we only used words for which we could identify and control for all the variables of interest described in the above-mentioned paragraph.

### Statistical analysis

We analysed data using JASP 0.16.1 (JASP Team 2022). First, we calculated the overall mean percentage of accuracy (percentage of correct yes/no responses) across the full experiment as well as the mean percentage of accuracy for each task individually to check that participants paid attention throughout. We obtained accuracy for the two internally oriented tasks by comparing responses during the main experiment with the responses to the post-task questionnaire. The answers to the questionnaire were the reference to which task responses had to coincide in order to be considered correct. We then used accuracy to decide what participants needed to be excluded for further analysis: i.e. we removed participant data if they failed to respond to ≥10% of the trials (cut-off based on pilot data from our lab) or if their accuracy levels for any of the four task conditions were <60%, as this would indicate they were not following the task instructions. As described in the Participants section, this resulted in the exclusion of 25 participants (final *n* = 200). Moreover, for each participant we removed outlier trials, defined by Tukey’s method (Tukey 1977) as RTs >1.5 interquartile ranges above the upper quartile (75%) or below the lower quartile (25%) for each of the 12 conditions (repetitions, within- and between-domain switches for each task) within each subject, as done in previous studies (Schmitz and Voss 2014; Verschooren *et al.* 2019b). This resulted in an average of 3.42% (±1.38) discarded trials per participant, in addition to the trials discarded due to incorrect responses (7.75% ± 4.76). We also removed trials with RTs of <200 ms (this occurred in six participants only; 2.31% ± 4.65 trials), as per previous studies (Arrington *et al.* 2003). This last step (removal of trials < 200 ms) was not specified in the pre-registration but was necessary to ensure that very fast (and likely meaningless) responses were excluded.

We then measured RTs for each trial type: repetitions and switches. We defined a ‘repetition’ trial as a trial in which participants performed the same task as in the previous trial. ‘Switch’ trials were trials in which participants performed a different task compared to the previous one. We further categorized switch trials as within- (internal-to-internal and external-to-external) or between-domain switches (from internal to external and vice versa).

We performed both frequentist and Bayesian statistics. We set the criterion for significance at *P *< 0.05 for frequentist statistics. For Bayesian tests, we always used default model priors (Cauchy distribution), and we considered a BF_10_ of ≥3 as substantial evidence for the alternate hypothesis (with BF_10_ ≥ 10 being strong evidence and a BF_10_ of ≥100 being very strong evidence) and a BF_10_ of ≤0.3 as substantial support for the null hypothesis, as per standard guidelines (Schönbrodt and Wagenmakers 2018). In cases of BF_10_ < 0.3, we report the BF_01_ instead, which quantifies the evidence in favour of the null hypothesis.

We performed all analyses on mean RTs for accurate trials only and excluded the data from the training phase. We followed any significant main effect or interaction in an ANOVA with *post hoc* pairwise *t*-tests using the Bonferroni correction for multiple comparisons.

We tested for normality but used ANOVAs even in cases where some violated these tests, since ANOVAs are robust enough even when assumptions of normality are not met (Schmider *et al.* 2010). Using Tukey’s method, we detected outlier participants and performed our pre-registered analyses both including and excluding these. In the next section, we report results that include data from all participants. Results after the removal of outlier participants are available in the Supplementary Materials section.

#### Pre-registered analyses

We performed a 2 × 2 repeated measures ANOVA (rANOVA) with domain (internal tasks and external tasks) and trial type (repetitions and switches) as factors to assess the presence and nature of switch costs in the two cognitive domains. Specifically, a main effect of trial type (followed by *post hoc* confirmation of higher RTs for switches compared to repetitions) would provide support for *Hypothesis 1*; a significant *post hoc t*-test revealing lower RTs for repetitions versus switches to an internal task would provide support for *Hypothesis 2* and an absence of interaction between domain and trial type would provide support for *Hypothesis 3*. Switches included all switch types (both within-domain and between-domain).

We then performed a 2 × 2 rANOVA with factors domain (internal tasks and external tasks) and switch type (within-domain and between-domain) on the switch costs. Switch costs were calculated by subtracting the average RTs during each switch type minus the average RTs during repetitions in the equivalent domains for each subject: e.g. switches from external to internal domain minus repetitions of internal tasks or switches within external domain minus repetitions of external tasks. This tested for *Hypothesis 4* (main effect of switch type with *post hoc* confirmation of higher RTs for between- versus within-domain switches) and our exploratory question about the direction of the between-domain switches.

A 1 × 4 rANOVA on RTs of repetition trials only (factor task repetition: External Task 1, External Task 2, Internal Task 1 and Internal Task 2) allowed us to check for the overall level of difficulty for each task (we expected to find no main effect), irrespective of switch costs.

Finally, we carried out a 4 × 2 rANOVA with factors task (External Task 1, External Task 2, Internal Task 1 and Internal Task 2) and switch type (within-domain and between-domain) on switch costs. For this, we calculated switch costs as the average RTs during switches minus the average RTs during repetitions for each task independently. For example, switches from both external tasks to Internal Task 1 (i.e. between-domain switches towards Internal Task 1) minus repetitions of Internal Task 1. This further investigated the potential effects of task difficulty when addressing *Hypothesis 4* and the exploratory question about the direction of the between-domain switches (Note that the above-mentioned 1 × 4 rANOVA highlighted an imbalance in task difficulty, as described in the Results section).

#### Exploratory analyses

In addition to the pre-registered analyses, to complement the 2 × 2 rANOVA on switch costs with factors domain and switch type, we performed one-sample *t*-tests comparing each switch type (switches within external and within internal domains, switches from internal to external and from external to internal) to 0. We performed this to make sure that the switch cost effects detected in our main analyses were not solely driven by between-domain switches (i.e. the most effortful ones). Therefore, this complements our main test for *Hypothesis 2* in the case of switches within the internal domain.

Similarly, to complement the 4 × 2 rANOVA on switch costs with factors task and switch type, we also performed one-sample *t*-tests comparing each switch type (within- and between-domain switches for each task) to 0 to check if they all significantly differed from repetitions (same rationale as mentioned earlier).

In addition to this, to account for the effect of task difficulty revealed in our pre-registered analyses, we grouped specific switch subtypes into three categories (hard-to-easy, neutral and easy-to-hard) based on results from the 1 × 4 ANOVA on task repetitions (see Fig. 4A). We then carried out a 1 × 3 rANOVA (frequentist and Bayesian) on switch costs with factor difficulty in the full sample to explore the possible influence of task difficulty, irrespective of domain and task. In grouping the switch subtypes (averaging across switch costs per participant), we interpreted switches from the consonant finding task (which had slower response times than the rest) to all other tasks as switches from a hard to an easy task. This was also the case for the switches from personality to current sensations tasks. In contrast, we interpreted switches from the vowel finding task to either internal task and vice versa as switches to tasks of similar difficulty (‘neutral’ switches). We considered all other switches (i.e. from all tasks towards consonant finding and from current sensations to personality) as switches from an easy to a hard task.

Moreover, to further assess whether our reported effects were influenced by differences in difficulty across tasks, we performed all our pre-registered analyses, as described earlier, also on a subset of 70 participants (mean age = 28.09 ± 4.02; 50 women and 20 men) that did not display a significant difference in difficulty between tasks (as indicated by the 1 × 4 rANOVA on task repetitions). With this approach, we aimed to completely exclude the influence of task difficulty on our effects of interest while still performing the specific analyses that we pre-registered. Specifically, this allowed us to confirm whether any differences (or similarities) between internal and external tasks were driven by differences in the difficulty of the tasks we used, or were inherent to the cognitive processes of interest, and thus aid the interpretation of our main results. We selected this subset of participants by randomly drawing 70 participants and performing the frequentist 1 × 4 rANOVA on task repetitions in an iterative fashion until non-significance was achieved.

Finally, at a reviewer’s suggestion, we performed an additional analysis to account for the effect of varied durations for task preparation. As explained in the Task section, our CTIs ranged between 200 and 6150 ms. In traditional task-switching paradigms (using externally oriented tasks), it is well established that the time allowed for preparation of the upcoming task (i.e. CTI) can greatly influence the magnitude of switch costs, with shorter CTIs leading to larger costs (Meiran 2000; Koch 2003; Kray 2006; Monsell and Mizon 2006; Kiesel *et al.* 2010). In order to establish whether this effect is also present in the internal domain and whether its influence is similar for internal than for external tasks, we divided our CTI distribution into three bins: short (200–1250 ms), medium (1600–3000 ms) and long (3350–6150 ms) CTI length. Note that each bin had a similar number of trials, although not identical due to the randomization. We then performed a 2 × 3 rANOVA with factors domain (internal and external) and CTI (short, medium and long) on switch costs. We also performed one-sample *t*-tests for each of the six switch cost conditions of this ANOVA to check whether switch costs were present across all CTI durations.

## Results

**Figure 2.**
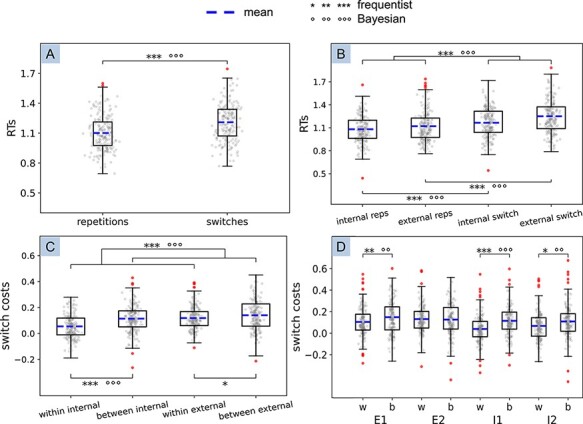
Boxplots representing data from all pre-registered statistical analyses. (**A**) Comparison of all repetitions versus all switches (overall switch costs); (**B**) main effect of trial type and *post hoc t*-tests from the 2 × 2 rANOVA with factors domain (internal and external) and trial type (switches and repetitions) that measures the switch costs in each domain; (**C**) main effect of switch type and *post hoc t*-tests from the 2 × 2 rANOVA on switch costs with factors domain and switch type (within- and between-domain) that measures the extra costs due to switching between domains; (**D**) *post hoc t*-tests from the 4 × 2 rANOVA on switch costs with factors task and switch type, aimed at further exploring the additional costs when switching across domains. Outlier participants appear as filled dots outside the upper and lower whiskers. These were included in all results reported in the main text (see the Supplementary Materials for results excluding outliers). The mean is represented as a dashed line. The whiskers represent the range between minimum and maximum, i.e. the variability outside the upper and lower quartiles. *P < 0.05, **P < 0.01, ***P < 0.001; °BF_10_ ≥ 3 (substantial evidence), °°BF_10_ ≥ 10 (strong evidence), °°°BF_10_ ≥ 100 (very strong evidence)

As mentioned in the Materials and Methods section, here we report results from all 200 participants, including outliers (see Fig. 2). Please refer to the Supplementary Materials section for the equivalent tests without outliers.

### Accuracy

Overall, participants completed the experiment with high accuracy (92.04% ± 5.19). The mean accuracy for the external tasks (consonant and vowel finding) was 94.39% (±5.46) and 94.92% (±5.66), respectively. Internal tasks (personality and current sensations) had a mean accuracy of 89.49% (±7.20) and 89.41% (±7.08), respectively.

### Switch costs

The Bayesian *t*-test comparing repetition and switch trials – performed as part of the Bayesian stopping rule – revealed very strong evidence for the presence of overall switch costs [BF_10_ = 5.763e + 52; frequentist *t*(199) = 22.27, *P *< 0.001, *d *= 1.57]. This provided support for *Hypothesis 1*. See Fig. 2A.

As can be expected, the 2 × 2 rANOVA with factors domain and trial type on RTs (Fig. 2B) also resulted in a significant main effect of trial type [BF_10_ = 4.337e + 34, *F*(1199) = 494.18, *P *< 0.001, $\eta _p^2$ = 0.71], with repetitions significantly faster than switches (*Hypothesis 1*). In addition, we found a significant main effect of domain indicating that, overall, external tasks led to slower RTs than internal tasks [BF_10_ = 8.061e + 9, *F*(1199) = 32.27, *P *< 0.001, $\eta _p^2$ = 0.14]. Finally, we found a significant interaction between domain and trial type [BF_10_ = 11.90, *F*(1199) = 37.13, *P *< 0.001, $\eta _p^2$ = 0.16], against our *Hypothesis 3*. As predicted, *post hoc t*-tests confirmed the presence of switch costs in both the external domain (BF_10_ = 1.836e + 46, *t *= 21.33, *P*_bonf_ < 0.001) and the internal domain (BF_10_ = 9.456e + 30, *t *= 13.91, *P*_bonf_ < 0.001), in support of *Hypothesis 2*. However, the difference between repetitions and switches was significantly greater in the external domain, as captured by the above-mentioned interaction. Table 1 displays means and standard deviations of all conditions.

**Table 1.** Mean and SD for all conditions in the pre-registered analyses. This includes all participants. All units are in seconds. E1: External Task 1 (consonant finding); E2: External Task 2 (vowel finding); I1: Internal Task 1 (personality task) and I2: Internal Task 2 (current sensations)

	Repetitions	Combined switches	Within-domain	Between-domain
All	1.102 (0.172)	1.209 (0.187)		
Internal	1.081 (0.178)	1.165 (0.187)	0.055 (0.094)	0.114 (0.103)
External	1.121 (0.200)	1.251 (0.219)	0.119 (0.094)	0.140 (0.121)
E1	1.165 (0.221)		0.106 (0.135)	0.150 (0.155)
E2	1.076 (0.197)		0.132 (0.132)	0.128 (0.153)
I1	1.097 (0.189)		0.041 (0.124)	0.116 (0.137)
I2	1.065 (0.181)		0.069 (0.133)	0.111 (0.147)

### Additional costs in between-domain switches

The 2 × 2 ANOVA with factors domain and switch type (Fig. 2C) on switch costs (i.e. RT for switch trials minus repetition trials) resulted in a significant main effect of domain [indicating slower responses for switches to external than to internal tasks; BF_10_ = 3.595e + 8, *F*(1199) = 37.03, *P *< 0.001, $\eta _p^2$ = 0.16] and switch type [BF_10_ = 4.761e + 6, *F*(1199) = 49.76, *P *< 0.001, $\eta _p^2$ = 0.20]. The latter supported *Hypothesis 4*, in showing additional costs when switching domains compared to staying within domain. However, we also found a significant interaction between domain and switch type [BF_10_ = 12.01, *F*(1199) = 10.81, *P *= 0.001, $\eta _p^2$ = 0.05]. *Post hoc t*-tests confirmed additional costs to switching from external to internal domain compared to staying within the internal domain (BF_10_ = 1.457e + 9, *t *= 7.31, *P*_bonf_ < 0.001). The extra costs in switches from the internal to external domain compared to those within the external domain were significant in our frequentist tests but showed only anecdotal Bayesian evidence (BF_10_ = 2.39, *t *= 2.67, *P*_bonf_ = 0.048).

Switching within the external domain resulted in higher RTs compared to switching within the internal domain (BF_10_ = 4.560e + 8, *t *= 6.82, *P*_bonf_ < 0.001). Moreover, switching from the internal to the external domain led to higher RTs compared to switching in the opposite direction, although with anecdotal Bayesian evidence (BF_10_ = 2.53, *t *= 2.79, *P*_bonf_ = 0.033). All four switch types exhibited a switch cost (i.e. switch cost different from 0; all one-sample *t*-tests: *P *< 0.001; see Supplementary Table S1A), including the switches within the internal domain (BF_10_ = 2.141e + 11, *t *= 8.20, *P *< 0.001, *d *= 0.58), further supporting *Hypothesis 2*.

Similarly, our 4 × 2 ANOVA comparing switch costs for each task individually revealed a significant main effect of task [BF_10_ = 1.207e + 7, *F*(3597) = 13.03, *P *< 0.001, $\eta _p^2$ = 0.06] and switch type [BF_10_ = 1.483e + 6, *F*(1199) = 44.18, *P *< 0.001, $\eta _p^2$ = 0.18], as well as a significant interaction [BF_10_ = 34.05, *F*(3597) = 8.03, *P *< 0.001, $\eta _p^2$ = 0.04] (Fig. 2D). *Post hoc t*-tests confirmed significantly greater costs in between-domain switches compared to within-domain ones for all tasks except vowel finding (Supplementary Table S2), explaining the significant interaction. All four tasks showed a switch cost (see Supplementary Table S1B). Table 1 displays means and standard deviations for all conditions.

### Difficulty of task repetitions

**Figure 3.**
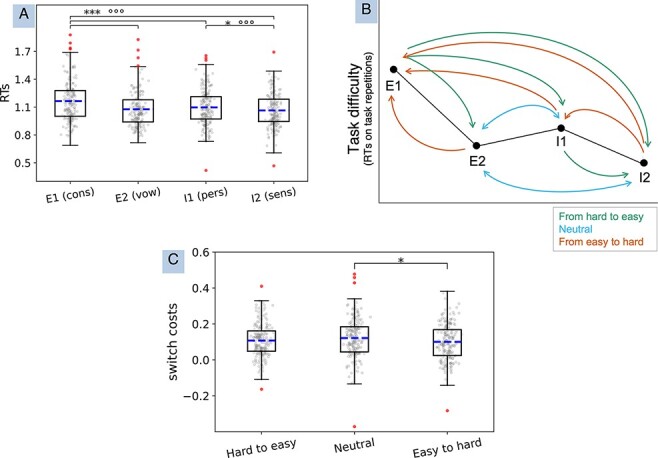
(**A**) 1 × 4 ANOVA with task repetitions to measure task difficulty, irrespective of switch costs. (**B**) Categorization of each switch subtype in the light of the level of difficulty of each task, as found in (A). (**C**) Boxplots showing the results from the 1 × 3 rANOVA (additional, non-pre-registered analysis) comparing switch costs grouped as hard-to-easy, neutral or easy-to-hard on the basis of differences in task difficulty. Outlier participants appear as filled dots outside the upper and lower whiskers (these were not removed from analyses). The mean is represented as a dashed line. The whiskers represent the range between minimum and maximum, i.e. the variability outside the upper and lower quartiles. *P < 0.05, **P < 0.01, ***P < 0.001; °BF_10_ ≥ 3 (substantial evidence), °°BF_10_ ≥10 (strong evidence), °°°BF_10_ ≥ 100 (very strong evidence)

We found a significant main effect in the 1 × 4 ANOVA (Fig. 3A; Supplementary Table S3) comparing RT for repetitions in each task [BF_10_ = 4.791e + 15, *F*(1.9,378.19) = 31.18, *P *< 0.001, $\eta _p^2$ = 0.135; the Greenhouse–Geisser sphericity correction was needed]. This suggests possible intrinsic differences in difficulty between task conditions, against our predictions. *Post hoc t*-tests indeed revealed that External Task 1 (consonant finding) resulted in higher RTs than all other tasks and that the personality task had higher RTs than the current sensations task (Supplementary Table S3). Table 1 includes the descriptive statistics of all conditions in the pre-registered analyses (RTs after subtraction of task repetitions are reported, where relevant; see Supplementary Table S4 for the raw RTs).

### Switch costs grouped by difficulty

We found no effect of difficulty in the 1 × 3 ANOVA on switch costs grouped according to the differences in task difficulty (i.e. from hard-to-easy, neutral and from easy-to-hard). However, there was a tendency towards significance with frequentist statistics only [hard-to-easy, 0.108 ± 0.094 s; neutral, 0.122 ± 0.109 s; easy-to-hard, 0.101 ± 0.100 s; BF_10_ = 0.329, *F*(1.879,373.874) = 3.03, *P *= 0.053, $\eta _p^2$ = 0.015; the Greenhouse–Geisser sphericity correction was needed]. This trend towards significance was likely led by the (weak) difference between neutral and easy-to-hard switches (neutral switches having larger switch costs; BF_10_ = 1.16, *t *= 2.42, *P*_bonf_ = 0.047), since no other *post hoc* comparison resulted significant (with hard-to-easy versus neutral having inconclusive evidence in either direction: BF_10_ = 0.404, *t *= 1.588, *P*_bonf_ = 0.339; hard-to-easy versus easy-to-hard having evidence in support of the lack of an effect: BF_01_ = 9.43, *t *= 0.84, *P*_bonf_ = 1.000). See Fig. 3 for a representation of the results.

### Subset of participants with no difference in task difficulty

**Figure 4.**
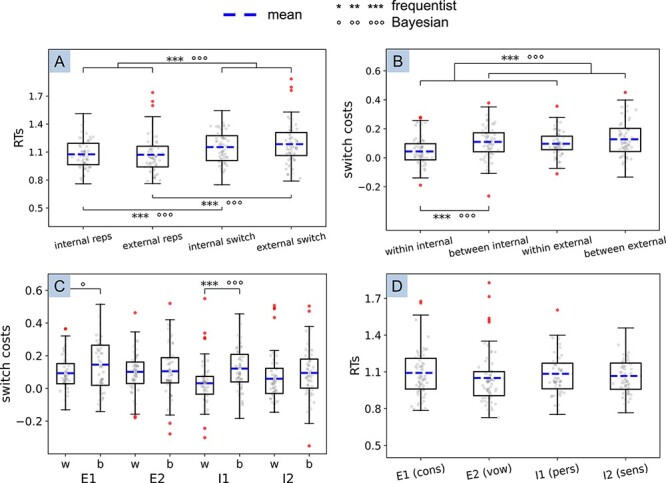
Boxplots showing data from a subset of 70 participants who did not show differences in task difficulty across the four tasks. (**A**) main effect of switch type and *post hoc t*-tests from the 2 × 2 rANOVA with factors domain and trial type (switch costs in each domain); (**B**) main effect of switch type and *post hoc t*-tests from the 2 × 2 rANOVA with factors domain and switch type (extra costs due to switching between domains); (**C**) 4 × 2 rANOVA with factors task and switch type (further exploration of additional costs when switching across domains); (**D**) 1 × 4 ANOVA with task repetitions (to measure task difficulty, irrespective of switch costs). Outlier participants appear as dots outside the upper and lower whiskers (these were not removed from analyses). The mean is represented as a dashed line. The whiskers represent the range between minimum and maximum, i.e. the variability outside the upper and lower quartiles. *P < 0.05, **P < 0.01, ***P < 0.001; °BF_10_ ≥ 3 (substantial evidence), °° BF_10_ ≥ 10 (strong evidence), °°°BF_10_ ≥ 100 (very strong evidence)

We analysed a subset of 70 participants that showed no significant difference in intrinsic task difficulty, i.e. no differences in RTs for repetitions across the four tasks [*F*(1.902,131.257) = 2.32, *P *= 0.105, $\eta _p^2$ = 0.03; with the Greenhouse–Geisser sphericity correction, Fig. 4D], along with strong Bayesian evidence supporting the absence of such difference (BF_01_ = 3.18). This replicated the majority of the effects described earlier for the main analyses, with a few differences. Specifically, the 2 × 2 ANOVA on RTs with factors domain and trial type (Fig. 4A) resulted in a significant main effect of trial type, which was consistent with our previous analyses [BF_10_ = 1.757e + 11, *F*(1,69) = 124.89, *P *< 0.001, $\eta _p^2$ = 0.64]. However, contrary to our above-mentioned results, we found no effect of domain [BF_01_ = 4.85, *F*(1,69) = 0.63, *P *= 0.43, $\eta _p^2$ = 0.009]. As mentioned earlier, the interaction was significant but now showed only inconclusive Bayesian evidence [BF_10_ = 0.48, *F*(1,69) = 7.49, *P *= 0.008, $\eta _p^2$ = 0.098]. As mentioned earlier, *post hoc* tests confirmed the presence of switch costs in both domains (external: BF_10_ = 1.723e + 13, *t *= 10.52, *P*_bonf_ < 0.001; internal: BF_10_ = 1.326e + 7, *t *= 7.18, *P*_bonf_ < 0.001).

Similarly, as mentioned earlier, the 2 × 2 ANOVA on switch costs with factors domain and switch type (Fig. 4B) returned a significant main effect of domain [BF_10_ = 10.60, *F*(1,69) = 7.33, *P *= 0.009, $\eta _p^2$ = 0.096] and switch type [BF_10_ = 607.25, *F*(1,69) = 23.72, *P *< 0.001, $\eta _p^2$ = 0.25] but no interaction [BF_10_ = 0.57, *F*(1,69) = 3.11, *P *= 0.082, $\eta _p^2$ = 0.04]. *Post hoc t*-tests revealed a significantly greater cost in switching from the external to internal domain versus switching within the internal domain, in accordance with the results in the full sample (BF_10_ = 2255.89, *t *= 4.68, *P*_bonf_ < 0.001), but there was no significant increase in switches from the internal to external domain compared to switches within the external domain (BF_10_ = 1.06, *t *= 2.18, *P*_bonf_ = 0.185). The difference between switches within internal and external domains remained significant (BF_10_ = 19.14, *t *= 3.22, *P*_bonf_ = 0.01), while the two types of between-domain switches were not significantly different anymore (BF_01_ = 4.53, *t *= 1.07, *P*_bonf_ = 1.00).

As mentioned earlier, the 4 × 2 ANOVA with factors task and switch type (Fig. 4C) revealed a main effect of task [with the Greenhouse–Geisser correction: *F*(2.688,185.489) = 3.172, *P *= 0.030, $\eta _p^2$ = 0.044] albeit with inconclusive Bayesian evidence (BF_10_= 0.93) and a main effect of switch type [BF_10_ = 375.14, *F*(1,69) = 19.01, *P *< 0.001, $\eta _p^2$ = 0.22]. Similarly, the interaction was significant as before [*F*(2.923,201.661) = 3.23, *P *= 0.024, $\eta _p^2$ = 0.045], although accompanied by inconclusive Bayesian evidence (BF_10_ = 0.58).

Table 2 includes the descriptive statistics of all conditions in these additional analyses (RTs after subtraction of task repetitions are reported, where relevant; see Supplementary Table S5 for the raw RTs).

### Effects of CTI duration

**Figure 5.**
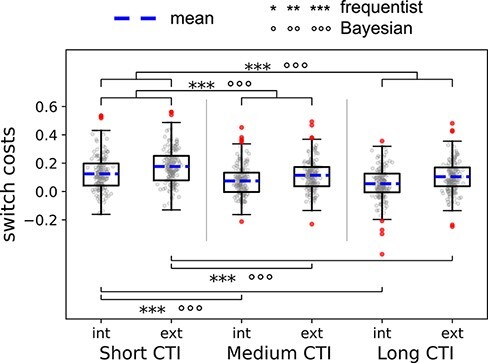
Boxplots representing the data from the analysis on the effects of CTI duration. The figure shows the *post hoc t*-tests from the 2 × 3 rANOVA on switch costs with factors domain (internal and external) and CTI (short, medium and long). Outlier participants appear as dots outside the upper and lower whiskers. These were included in all results reported in the main text (see the Supplementary Materials for results excluding outliers). The mean is represented as a dashed line. The whiskers represent the range between minimum and maximum, i.e. the variability outside the upper and lower quartiles. *P < 0.05, **P < 0.01, ***P < 0.001; °BF_10_ ≥ 3 (substantial evidence), °°BF_10_ ≥ 10 (strong evidence), °°°BF_10_ ≥ 100 (very strong evidence)

The 2 × 3 rANOVA on switch costs with factors domain and CTI (Fig. 5) showed a significant main effect of CTI [BF_10_ = 6.912e + 17, *F*(1.908,379.715) = 59.01, *P *< 0.001, $\eta _p^2$ = 0.23; with the Greenhouse–Geisser sphericity correction] and domain [with greater switch costs in the external as compared to internal domain; BF_10_ = 3.010e + 10, *F*(1199) = 39.93, *P *< 0.001, $\eta _p^2$ = 0.17], but strong evidence for the lack of an interaction [BF_01_ = 38.07, *F*(1.913,380.732) = 0.44, *P *= 0.634, $\eta _p^2$ = 0.002; with the Greenhouse–Geisser sphericity correction]. *Post hoc t*-tests revealed greater switch costs in short CTIs compared to both medium (BF_10_ = 4.193e + 12, *t *= 8.16, *P*_bonf_ < 0.001) and long CTIs (BF_10_ = 3.739e + 18, *t *= 10.29, *P*_bonf_ < 0.001) but no difference between medium and long CTIs (BF_10_ = 0.53, *t *= 2.14, *P*_bonf_ = 0.10). This pattern was present both in the internal (short versus medium: BF_10_ = 6975.18, *t *= 5.16, *P*_bonf_ < 0.001; short versus long: BF_10_ = 2.187e + 8, *t *= 7.16, *P*_bonf_ < 0.001; medium versus long: BF_10_ = 0.48, *t *= 2.01, *P*_bonf_ = 0.67) and external domains (short versus medium: BF_10_ = 2.273e + 8, *t *= 6.42, *P*_bonf_ < 0.001; short versus long: BF_10_ = 1.306e + 9, *t *= 7.44, *P*_bonf_ < 0.001; medium versus long: BF_01_ = 7.25, *t *= 1.02, *P*_bonf_ = 1). Importantly, despite the difference between short and medium/long CTIs, switch costs were significantly >0 in all CTI durations, as measured by the one-sample *t*-tests (all *P *< 0.001). Table 3 includes the descriptive statistics of all conditions in this analysis (RTs after subtraction of task repetitions are reported; see Table S6 for the raw RTs).

## Discussion

In this study, we tested a novel paradigm for the investigation of task switches within and between externally and internally oriented (specifically self-referential) cognitive domains. We provide the first evidence of switch costs towards self-referential tasks, confirming our main hypothesis. Interestingly and contrary to what we expected, internal switch costs were smaller than those between our external tasks (which we designed to be similar to the tasks more typically studied in the task-switching literature). Crucially, we also observed an additional cost for switches across the two domains (internal to external and vice versa) compared to within-domain switches, confirming our fourth hypothesis.

**Table 2.** Descriptive statistics of all conditions in the analyses of a balanced subset of 70 participants. E1: External Task 1 (consonant finding); E2: External Task 2 (vowel finding); I1: Internal Task 1 (personality task) and I2: Internal Task 2 (current sensations)

	Repetitions	Combined switches	Within-domain	Between-domain
All	1.074 (0.164)	1.169 (0.181)		
Internal	1.075 (0.155)	1.153 (0.173)	0.044 (0.102)	0.110 (0.112)
External	1.071 (0.199)	1.184 (0.217)	0.097 (0.088)	0.128 (0.125)
E1	1.092 (0.195)		0.093 (0.109)	0.145 (0.160)
E2	1.050 (0.216)		0.101 (0.119)	0.105 (0.166)
I1	1.084 (0.169)		0.031 (0.131)	0.121 (0.131)
I2	1.067 (0.154)		0.059 (0.142)	0.095 (0.150)

There is vast literature reporting costs associated with switching between externally oriented tasks [see Monsell (2003), Kiesel *et al.* (2010) and Vandierendonck *et al.* (2010) for reviews]. Here, we found evidence of similar switch costs in the internal, self-referential domain as well. Importantly, these costs were present when taking into account all types of switches towards internal tasks (i.e. both those coming from an internal or from an external task), but also when focusing only on switches within the internal domain. To our knowledge, this is the first study to report switch cost effects involving self-referential processes. In an earlier study aimed at assessing the impact of mindfulness meditation over cognitive control, Chambers *et al.* (2008) used an attention-switching task that included blocks of neutral (food/household objects) versus affective (positive/negative) words with the aim of measuring ‘internal switching’ on affective stimuli. However, their paradigm instructed participants to mentally count the appearance of each word category across the task and, therefore, while the nature of the stimuli (neutral versus affective) could be described as external versus internal (respectively), the task itself involved mental counting for both types of words, rather than an actual affective or, in our case, self-referential judgement. In that sense, the underlying processes in their study are similar to those captured by traditional task-switching studies employing working memory tasks.

**Table 3.** Mean and SD for all conditions in the 2 × 3 rANOVA. This includes all participants. All units are in seconds

	Internal	External
Short CTIs	0.125 (0.125)	0.177 (0.132)
Medium CTIs	0.075 (0.107)	0.114 (0.111)
Long CTIs	0.055 (0.112)	0.104 (0.112)

The presence of switch costs both in the internal and external domains in our data could be explained by either the presence of one domain-general mechanism for task-switching that controls both domains (as suggested by some studies; see, e.g. Chiu and Yantis 2009) or the existence of distinct domain-specific mechanisms that operate in a similar fashion. Importantly and contrary to what we expected (see *Hypothesis 3*), we found a significant interaction between domain and trial type, whereby switch costs were greater for external than for internal tasks. While, in our main analyses, this interaction could have been partially due to a difficulty imbalance across the tasks, the same analysis over a subset of participants that showed no significant differences in the RTs of task repetitions still returned a significant effect (albeit with much weaker Bayesian evidence). This might suggest the presence of two distinct (domain-specific) mechanisms for the flexible control of internal and external processes, whereby the difference in the magnitude of switch costs would reflect differences in the underlying cognitive processes (i.e. in the way goal-shifting and rule retrieval and activation are implemented). In line with this view, a number of studies point towards switch costs capturing an increased preparatory control effort (during switches compared to repetitions) in the system responsible for the task, instead of a dedicated process in place for task-switching specifically (Kiesel *et al.* 2010; Ruge *et al.* 2013). For instance, studies that systematically varied the CTI showed benefits of longer preparation time (leading to faster responses) not only on switch trials but on repetition trials as well (Altmann 2004). Similarly, the predictability of the upcoming task appears to benefit the preparation of both switches and repetitions, indicating that task-specific control processes are engaged both when switching and repeating tasks (Dreisbach *et al.* 2002; Koch 2005). Furthermore, neuroimaging research has identified a set of regions typically reported as belonging to the dorsal attention network (DAN; i.e. inferior frontal gyrus, inferior frontal junction, pre-supplementary motor area (pre-SMA), intraparietal sulcus and posterior parietal cortex) that appear more active during switches between similar (external) tasks compared to repetitions (Braver *et al.* 2003; Brass and Cramon von 2004). Interestingly, however, most of these regions also appeared to activate during task repetitions (Dove *et al.* 2000), providing evidence against a specific network dedicated to task-set reconfiguration during switches only and in support of an effect of an adaptation or facilitation process during task repetitions instead (i.e. leading to less activation in the network during repetitions; De Baene *et al.* 2012). Similarly, studies employing tasks that rely on different processes (e.g. colour versus motion discrimination and semantic versus spatial tasks) showed activation of lower-level areas (e.g. fusiform gyrus for colour encoding) and differential ERP signatures that depend on the specific task features during the post-cue preparatory period of switch trials versus repetitions (Wylie *et al.* 2006; Capizzi *et al.* 2015). These results possibly suggest the presence of varied subtypes of preparatory control (or at least a dynamic interaction between general control areas and other more specialized areas) that can be engaged depending on the specific task demands (Ruge *et al.* 2013). While behavioural evidence cannot directly speak about underlying networks, our results seem to agree with this view of two local, specific systems that aid the flexible control of self-referential and external processes, respectively.

Crucially, we also found additional costs associated with switches across the two domains compared to those that stayed within-domain, although this effect was weaker in switches towards the external domain (i.e. we only obtained anecdotal Bayesian evidence when comparing internal-to-external switches versus within-external switches). Our results are in apparent contrast to Verschooren *et al.* (2019b) who found that switch costs between perceptual and working memory tasks were similar to those within the perceptual domain. The authors proposed that their results support the presence of one limited shared resource that the two domains (perception and memory) have to compete for (termed ‘resource sharing account’; Gilbert *et al.* 2005; Burgess *et al.* 2007). As discussed earlier on, they considered working memory as a separate domain in that (unlike perception and other classically used tasks in the task-switching literature) it entails a focus on information that is not perceptually available (versus stimulus-dependent information). However, the inherent differences with self-referential processes preclude direct comparisons between our results. Self-referential cognition is defined by a focus on information about the self as opposed to information about the external world (i.e. a distinction in the content of information regardless of perceptual input). In contrast, working memory would be considered part of our definition of external domain due to its focus on non-self stimuli and its engagement of lateral frontoparietal regions (Pollmann and von Cramon 2000; Awh and Jonides 2001). The resource sharing account thus may provide an explanation for the control of externally oriented (perceptual or working memory) processes but is not exhaustive for the interpretation of our findings that include the self-referential domain.

Instead, we believe that the additional cost between domains in our study might reflect a more effortful control needed to switch between different domains, as the tasks are subserved by different (domain-specific) cognitive systems, compared to the effort and resources needed to switch within the same system configuration. In other words, the necessary steps of deactivating the currently irrelevant system and activating the relevant one would take more time compared to more ‘local’ within-domain adjustments. To allow an effective communication between domain-specific systems, one possibility is that these systems are directly linked with each other. As discussed earlier, regions of the DAN show increased activation during task switches. In contrast, the default mode network (DMN) has been involved in the performance of self-referential tasks similar to the ones we used in our study (see Kelley *et al.* 2002; Davey *et al.* 2016) but is also recruited by large shifts in cognitive context (Crittenden *et al.* 2015; Smith *et al.* 2019), as well as in the preparation of rest trials (Sidlauskaite *et al.* 2014) in other task-switching paradigms. It is thus possible that the DMN would link with the DAN, possibly via a functional segregation of the precuneus (Leech *et al.* 2011). Indeed, these two networks are known to anti-correlate both at rest (Fox *et al.* 2005) and during cognitive tasks (Weissman *et al.* 2006; Kelly *et al.* 2008). Another possibility is that switching across domains engages a higher-level domain-general control system, which couples with both specific subsystems when a domain shifting is needed. In the task-switching literature, there is evidence for the concurrent engagement of a small number of DAN areas (including the superior parietal cortex and anterior cingulate/pre-SMA) during switching, irrespective of specific task features and switch types. In contrast, other regions in this network show task-specific modulations (Yeung *et al.* 2006; Esterman *et al.* 2009; Tamber-Rosenau *et al.* 2011; Kim *et al.* 2012). This could suggest a higher-order control system that can coordinate the implementation of task-relevant rules during cognitive shifts (Yeung *et al.* 2006). Importantly, however, this was found through studies that employed externally oriented tasks only. In contrast, a vast literature on the dynamics of large-scale intrinsic networks points towards a role of the salience network in the flexible coupling of DMN and DAN to support adaptive changes between external and internal processes (Sridharan *et al.* 2008; Spreng *et al.* 2010; Shaw *et al.* 2021), suggesting that this may be a potential candidate to regulate between domain (internal/external) switches. Future neuroimaging studies may clarify whether this is the case.

Importantly, we found that the magnitude of the switch costs in our paradigm was influenced by the length of preparation time (CTI duration) in a similar way for internal and external tasks. Specifically, short CTIs induced greater switch costs than medium and long ones in both domains. This is consistent with the vast literature demonstrating that longer preparation times have a facilitatory effect on task-switching for externally oriented tasks (Koch 2003; Monsell and Mizon 2006; Kiesel *et al.* 2010), and expands those findings to the internal domain for the first time. Crucially, the lack of interactions between CTI duration and domain suggests that the internal and external cognitive systems might share common features of cognitive control, further supporting the above-discussed presence of domain-general processes, affecting the preparatory phase in this case. It is important to also note that both domains retained a switch-cost effect even after long preparatory times, consistently with previous studies using our CTI jittering (De Baene and Brass 2011, 2013; Sidlauskaite *et al.* 2014) and further supporting the suitability of our design for the study of task-switching.

In a similar line, we found an interaction between domain and switch type, further suggesting that the extra costs associated with between-domain switching differed depending on which domain participants switched from and to. *Post hoc* comparisons revealed that this interaction was mainly led by a marked difference among the within-domain switches. Specifically, switching within the external domain induced slower RTs compared to switching within the internal domain. Furthermore, while this difference persisted in the subsample of participants with a balanced difficulty across tasks, the between-domain asymmetry did not. It is possible that the slower RTs in external within-domain switches in the full group were (at least partially) induced by the large difference in difficulty between Tasks E1 and E2. Previous studies showed that an imbalanced dominance and/or familiarity between tasks can influence the magnitude of switch costs, with higher costs related to switching to the more dominant task compared to switching to the less dominant one. For instance, word reading was associated with greater costs compared to colour naming in the Stroop task (Yeung and Monsell 2003; Wu *et al.* 2015). However, while according to this logic, switching from Task E1 (harder) to Task E2 (easier) should result in slower RTs compared to the opposite switch, such a difference was not present in our data. However, the wide difference between switches within the internal and external domains was only reduced (not abolished) in the balanced sample of participants, indicating that task difficulty was not the (only) cause for the interaction between domain and switch type in our data. This further supports the idea that the magnitude of the switch costs within each domain might reflect the employment of domain-specific cognitive processes (as stated earlier). It is also possible that our experimental design induced a stronger task-set interference between external tasks (sharing similar instructions and stimuli lists) compared to between internal tasks (where tasks require larger shifts in focus and have homogeneous lists of either trait or bodily adjectives) due to a higher level of competition between the stimuli features (Sdoia and Ferlazzo 2008). However, it is worth noting that, because the bodily adjectives did not all relate to purely physical sensations (e.g. ‘hungry’) but also included emotional states that are accompanied by bodily sensations (e.g. ‘anxious’), the difference between these words and those used for the personality task might not have been obvious to participants and thus should have minimized this issue. In either case, studies that systematically varied the number of stimuli and response modalities (Dykstra *et al.* 2022) or included switching between similar and dissimilar tasks (Arrington *et al.* 2003; Crittenden *et al.* 2015) and domains (Foti *et al.* 2015) highlighted that, in agreement with our results, the specific task demands and characteristics do influence switch costs.

There are a number of further considerations to be acknowledged when interpreting our data. First, we had to rely on a proxy measure for the accuracy of the internal conditions (via a post-task questionnaire), as the required judgements inherently lack a ‘ground truth’ that can be directly accessed by other than the participants themselves. This is naturally a less objective and reliable measure as compared to our external tasks. Without a ground truth measure, it is not possible to establish whether our participants were truly able to accurately assess information about themselves. However, while we used self-referential tasks in a task-switching paradigm for the first time, personality judgement tasks have been extensively used in previous studies on the sense of self and its neural bases in similarly fast-paced experiments (Kelley *et al.* 2002; D’Argembeau *et al.* 2007; Davey *et al.* 2016). Moreover, where accuracy was lower for internal than for external tasks, it remained very high (and with low variance) across them (>89%), with internal tasks displaying faster or similar RTs to the external ones. Overall, this suggests that our participants were indeed able to successfully perform the self-referential tasks. Moreover, we focused our analysis on RTs only, and only included trials with correct responses. Therefore, any potential influence of the discrepancy between internal and external tasks should be limited. In either case, we used accuracy to make sure that participants were following instructions and engaging in the processes of interest and, in this case, attempting to access internal information. Understanding whether they were reliable and truthful in their personality assessment or measuring their interoceptive sensibility was beyond the scope of our study and remains an interesting question to be addressed in future research.

Second, our externally oriented tasks required the detection and categorization of specific letters in a written word, thus calling for a shift in spatial attention (to locate the third or penultimate letter) and orthographic classification (to recognize consonants or vowels). In contrast, the internally oriented tasks both required a more conceptual (semantic) encoding of the words, as well as autobiographical knowledge (personality task) and interoceptive sensibility (current sensations task). Therefore, the level of stimulus processing required by each domain is different. Previous studies reported greater switch costs for switches between dissimilar tasks compared to switches between tasks that share the same cognitive operations (Arrington *et al.* 2003). For instance, switching within two similar language tasks, within two semantic categorization tasks or within visual perception tasks was found to be easier (faster RTs) than switching across them (Crittenden *et al.* 2015). Similarly, the switch costs associated with switching within attention to the form of visual stimuli (width and height) or within attention to their colour (hue and brightness) were smaller compared to the costs of switching between those perceptual features (Arrington *et al.* 2003). Importantly, this similarity effect was not related or attributable to differences in the difficulty of specific tasks. While this did not affect the presence of reliable switch costs in our paradigm (which we found in both domains even when only taking within-domain switches into account), it might have influenced over the magnitude of the additional costs we found in between-domain versus within-domain switches. It is however important to note that these differences are intrinsic to the specific processes we aimed to investigate and thus were not avoidable. As stated elsewhere in this article, we designed our external tasks to bridge with the vast task-switching literature employing digit and letter categorization (Rogers and Monsell 1995; Schneider and Logan 2010) while keeping the same stimuli for both domains. Future studies might focus on testing this paradigm with other (more semantic) externally oriented tasks.

## Conclusions

We identified switch costs in both external and internal (self-referential) domains and an additional cost for between-domain versus within-domain switches. This may suggest the goal-directed engagement of two domain-specific cognitive systems (for the control of external and internal processes) that communicate and share domain-general control features for their flexible management.

## Supplementary Material

niac016_Supp

## Data Availability

Data available on OSF at https://osf.io/jnxd2/?view_only=33fd2662d5e842289c8119aff55f2e04.
