# Supplementary material for: Investigating the shift between externally and internally oriented cognition: a novel task-switching paradigm
Source: Neurosci Conscious. 2022 Nov 19;2022(1):niac016. doi: 10.1093/nc/niac016 (PMC9675616; doi:10.1093/nc/niac016)
Supplement: niac016_Supp [file niac016_supp.zip › suppl_data/Supplementary_Materials_Calzolari_Boneva_Fernandez-Espejo_revised_clean.docx]

**8. Supplementary Materials**

**8.1 Additional result tables**

|  |  | **frequentist** | | | | **Bayesian** |
| --- | --- | --- | --- | --- | --- | --- |
|  |  | **t** | **df** | **p** | **Cohen’s d** | **BF_10_** |
| *A* | **within internal** | 8.203 | 199 | < .001 | 0.580 | 2.141e+11 |
|  | **between internal** | 15.612 |  | < .001 | 1.104 | 1.251e+33 |
|  | **within external** | 17.867 |  | < .001 | 1.263 | 7.059e+39 |
|  | **between external** | 16.432 |  | < .001 | 1.162 | 3.720e+35 |
| *B* | **E1, within** | 11.102 | 199 | < .001 | 0.785 | 6.195e+19 |
|  | **E1, between** | 13.687 |  | < .001 | 0.968 | 3.614e+27 |
|  | **E2, within** | 14.235 |  | < .001 | 1.007 | 1.668e+29 |
|  | **E2, between** | 11.826 |  | < .001 | 0.836 | 8.733e+21 |
|  | **I1, within** | 4.624 |  | < .001 | 0.327 | 3543.073 |
|  | **I1, between** | 12.001 |  | < .001 | 0.849 | 2.929e+22 |
|  | **I2, within** | 7.353 |  | < .001 | 0.520 | 2.851e+9 |
|  | **I2, between** | 10.600 |  | < .001 | 0.750 | 2.091e+18 |

Table S1. One sample t-tests over switch types after subtracting repetitions to check that all switch types in the 2x2 (A) and 4x2 (B) rANOVAs were significantly different from task repetitions (=0). Includes all participants.

|  | | | **frequentist** | | **Bayesian** |
| --- | --- | --- | --- | --- | --- |
|  | | | **t** | **p_bonf_** | **BF_10_** |
| **E1, within** | **-** | **E1, between**** | -3.766 | 0.005 | 51.150 |
| **E2, within** | **-** | **E2, between**** | 0.385 | 1.000 | 0.085 |
| **I1, within** | **-** | **I1, between**** | -6.480 | < .001 | 1.257e+7 |
| **I2, within** | **-** | **I2, between**** | -3.546 | 0.012 | 29.122 |
| ** Frequentist p-value adjusted for comparing a family of 28 | | | | | |

Table S2. Post-hoc t-tests (frequentist and Bayesian) from the 4x2 repeated measures ANOVA with factors task and switch type performed on switch costs (with subtraction of task repetitions), Includes all participants. E1: external task 1 (consonant finding); E2: external task 2 (vowel finding); I1: internal task 1 (personality task); I2: internal task 2 (current sensations).

|  | | | **frequentist** | | **Bayesian** |
| --- | --- | --- | --- | --- | --- |
|  | | | **t** | **p_bonf_** | **BF_10_** |
| **E1/consonants** | **-** | **E2/vowels** | 7.787 | < .001 | 1.637e+17 |
|  |  | **I1/personality** | 5.986 | < .001 | 6.993.178 |
|  |  | **I2/sensations** | 8.840 | < .001 | 2.296e+9 |
| **E2/vowels** | **-** | **I1/personality** | -1.801 | 0.433 | 0.335 |
|  |  | **I2/sensations** | 1.053 | 1.000 | 0.133 |
| **I1/personality** | **-** | **I2/sensations** | 2.854 | 0.027 | 1077.291 |
| *^ᵃ^ Greenhouse-Geisser correction was applied due to violated assumption of sphericity (p < .05)*  *Note.* Frequentist p-value adjusted for comparing a family of 6 (*) | | | | | |

Table S3. Post-hoc t-tests from the 1x4 repeated measures ANOVA (frequentist and Bayesian) on task repetitions. This was used to check whether there was an intrinsic difference in task difficulty, irrespective of switch costs. Includes all participants.

|  | **Within domain** | **Between domains** | **Hard-to-easy switches** | **Neutral switches** | **Easy-to-hard switches** |
| --- | --- | --- | --- | --- | --- |
|  |  |  | 1.179 (0.198) | 1.199 (0.192) | 1.243 (0.203) |
| **Internal** | 1.135 (0.190) | 1.195 (0.201) |  |  |  |
| **External** | 1.239 (0.221) | 1.261 (0.232) |  |  |  |
| **E1** | 1.271 (0.243) | 1.315 (0.261) |  |  |  |
| **E2** | 1.209 (0.238) | 1.204 (0.237) |  |  |  |
| **I1** | 1.137 (0.208) | 1.213 (0.216) |  |  |  |
| **I2** | 1.133 (0.208) | 1.175 (0.227) |  |  |  |

Table S4. Descriptive statistics of RTs for all conditions in the 2x2 ANOVA (domain x switch type), 4x2 ANOVA (task x switch type) and 1x3 ANOVA (difficulty) on switch costs. Includes all participants. E1: external task 1 (consonant finding); E2: external task 2 (vowel finding); I1: internal task 1 (personality task); I2: internal task 2 (current sensations).

|  | **Within domain** | **Between domains** |
| --- | --- | --- |
| **Internal** | 1.119 (0.176) | 1.185 (0.187) |
| **External** | 1.168 (0.214) | 1.199 (0.237) |
| **E1** | 1.185 (0.224) | 1.238 (0.260) |
| **E2** | 1.151 (0.229) | 1.155 (0.247) |
| **I1** | 1.116 (0.190) | 1.206 (0.207) |
| **I2** | 1.126 (0.212) | 1.162 (0.202) |

Table S5. Descriptive statistics of RTs for all conditions in the 2x2 (domain x switch type) and 4x2 ANOVAs (task x switch type) when analysing a balanced subset of 70 participants. E1: external task 1 (consonant finding); E2: external task 2 (vowel finding); I1: internal task 1 (personality task); I2: internal task 2 (current sensations).

|  |  | **Internal** | **External** |  |
| --- | --- | --- | --- | --- |
| **Short CTIs** | **Repetitions** | 1.090 (0.187) | 1.143 (0.213) | |
|  | **Switches** | 1.215 (0.219) | 1.319 (0.259) | |
| **Medium CTIs** | **Repetitions** | 1.071 (0.183) | 1.099 (0.194) | |
|  | **Switches** | 1.146 (0.195) | 1.214 (0.213) | |
| **Long CTIs** | **Repetitions** | 1.082 (0.185) | 1.118 (0.208) | |
|  | **Switches** | 1.137 (0.189) | 1.223 (0.228) | |

Table S6. Descriptive statistics of RTs for all conditions in the 2x3 (domain x CTI) ANOVA. E1: external task 1 (consonant finding); E2: external task 2 (vowel finding); I1: internal task 1 (personality task); I2: internal task 2 (current sensations).

|  |  | **frequentist** | | | | **Bayesian** |
| --- | --- | --- | --- | --- | --- | --- |
|  |  | **t** | **df** | **p** | **Cohen’s d** | **BF_10_** |
|  | **internal switches – short CTIs** | 14.050 | 199 | < .001 | 0.993 | 2.284e+28 |
|  | **internal switches – medium CTIs** | 9.837 |  | < .001 | 0.696 | 6.613e+15 |
|  | **internal switches – long CTIs** | 6.930 |  | < .001 | 0.490 | 1.323e+8 |
|  | **external switches – short CTIs** | 18.875 |  | < .001 | 1.335 | 6.443e+42 |
|  | **external switches – medium CTIs** | 14.537 |  | < .001 | 1.028 | 6.888e+29 |
|  | **external switches – long CTIs** | 13.161 |  | < .001 | 0.931 | 4.592e+25 |

Table S7. One sample t-tests over internal and external switches in each CTI condition (after subtracting repetitions) to check that all switch costs in the 2x3 rANOVA were significantly different from task repetitions (=0). Includes all participants.

**8.2 Statistical analyses after removing outlier participants**

*8.2.1 Switch costs*

|  | | | **frequentist** | | | | **Bayesian** |
| --- | --- | --- | --- | --- | --- | --- | --- |
|  | | | **t** | **df** | **p** | **Cohen's d** | **BF_10_** |
| **repetitions**  ***(****1.097±0.166****)*** | - | **switches**  (*1.204±0.181)* | -22.117 | 197 | < .001 | -1.572 | 1.227e+52 |
| *Note.* The alternative hypothesis specifies that repeats is smaller than switches. | | | | | | | |

Table **S8**. Paired Samples T-test (frequentist and Bayesian) for finding overall switch costs (all repetitions versus all switches), when excluding 2 outlier participants.

|  | **frequentist** | | | | | **Bayesian** |
| --- | --- | --- | --- | --- | --- | --- |
|  |  | **df** | **F** | **p** | **η² _p_** | **BF_10_** |
| **domain** |  | 1,191 | 24.020 | < .001 | 0.112 | 3.254e+60 |
| **trial type** |  | 1,191 | 482.533 | < .001 | 0.716 | 7.059e+38 |
| **domain ✻ trial type** | | 1,191 | 40.452 | < .001 | 0.175 | 27.642 |
|  | | |  | **t** | **p_bonf_** | **BF_10_** |
| **internal**  ***(****1.118±0.167****)*** | **-** | **external**  *(1.168±0.184)* |  | -4.901 | < .001 | 1.918e+7 |
| **repetitions**  ***(****1.097±0.166****)*** | - | **switches**  (*1.204±0.181)* |  | -21.967 | < .001 | 3.932e+74 |
| **int. repeat.**  ***(****1.076±0.166)* | **-** | **ext. repeat.***  ***(****1.103±0.176)* |  | -2.462 | 0.087 | 2.010 |
|  |  | **int. switch.***  ***(****1.160±0.177)* |  | -13.747 | < .001 | 3.689e+29 |
|  |  | **ext. switch.***  ***(****1.234±0.202)* |  | -13.901 | < .001 | 5.043e+25 |
| **ext. repeat.**  ***(****1.103±0.176)* | **-** | **int. switch.***  ***(****1.160±0.177)* |  | -5.059 | < .001 | 36893.923 |
|  |  | **ext. switch.***  ***(****1.234±0.202)* |  | -21.385 | < .001 | 1.336e+46 |
| **int. switch.**  ***(****1.160±0.177)* | **-** | **ext. switch.***  ***(****1.234±0.202)* |  | -6.763 | < .001 | 1.133e+7 |
| *Note.* Frequentist p-value adjusted for comparing a family of 6 (*)  Underlined comparisons signal a change in significance with respect to the analysis with all participants  (int rep vs ext rep including all participants: *p* = .004) | | | | | | |

Table **S9**. 2x2 repeated measures ANOVA (frequentist and Bayesian) with factors domain and trial type, to assess the presence and nature of switch costs in the two cognitive domains. 8 outlier participants were excluded.

*8.2.2 Additional costs in between-domain switches*

|  | **frequentist** | | | | | **Bayesian** |
| --- | --- | --- | --- | --- | --- | --- |
|  |  | **df** | **F** | **p** | **η² _p_** | **BF_10_** |
| **domain** |  | 1,186 | 32.696 | < .001 | 0.150 | 2.893e+7 |
| **switch type** |  | 1,186 | 52.976 | < .001 | 0.222 | 1.838e+7 |
| **domain ✻ switch type** |  | 1,186 | 10.587 | 0.001 | 0.054 | 8.175 |
|  | | |  | **t** | **p_bonf_** | **BF_10_** |
| **internal**  ***(****0.083±0.075)* | **-** | **external**  ***(****0.125±0.079)* |  | -5.718 | < .001 | 9.485e+6 |
| **within**  ***(****0.083±0.063)* | **-** | **between**  ***(****0.125±0.077)* |  | -7.278 | < .001 | 1.623e+9 |
| **within int.**  ***(****0.053±0.092)* | **-** | **within ext.***  ***(****0.113±0.080)* |  | -6.513 | < .001 | 2.045e+8 |
|  |  | **between int.***  ***(****0.113±0.092)* |  | **-7.450** | **< .001** | **1.081e+10** |
| **within ext.**  ***(****0.113±0.080)* | **-** | **between ext.***  ***(****0.136±0.112)* |  | **-2.853** | **0.027** | **3.182** |
| **between int.**  ***(****0.113±0.092)* | **-** | **between ext.***  ***(****0.136±0.112)* |  | -2.527 | 0.072 | 1.231 |
| *Note.* Frequentist p-value adjusted for comparing a family of 6 (*)  Underlined comparisons signal a change in significance with respect to the analysis with all participants | | | | | | |

Table **S10**. 2x2 repeated measures ANOVA (frequentist and Bayesian) with factors domain and switch type performed on the switch costs (mean switches after subtraction of mean repetitions for each subject), to explore the extra costs of switching across domains versus switching while staying within the same domain. 13 outlier participants were excluded.

|  | | | **frequentist** | | | **Bayesian** |
| --- | --- | --- | --- | --- | --- | --- |
|  |  | **df** | **F** | **p** | **η² _p_** | **BF_10_** |
| **task ^a^** |  | 2.858,462.959 | 17.775 | < .001 | 0.099 | 6.609e+9 |
| **switch type** |  | 1,162 | 56.404 | < .001 | 0.258 | 6.602e+8 |
| **task ✻ switch type** |  | 2.974,481.733 | 7.152 | < .001 | 0.042 | 21.176 |
|  | | |  | **t** | **p_bonf_** | **BF_10_** |
| **E1**  ***(****0.120±0.106)* | **-** | **E2***  ***(****0.129±0.101)* |  | -0.936 | 1.000 | 0.105 |
|  |  | **I1***  ***(****0.073±0.085)* |  | 4.913 | < .001 | 17773.654 |
|  |  | **I2***  ***(****0.078±0.090)* |  | 4.364 | < .001 | 1257.844 |
| **E2**  ***(****0.129±0.101)* | **-** | **I1***  ***(****0.073±0.085)* |  | 5.849 | < .001 | 3.475e+6 |
|  |  | **I2***  ***(****0.078±0.090)* |  | 5.299 | < .001 | 362652.970 |
| **I1**  ***(****0.073±0.085)* | **-** | **I2***  ***(****0.078±0.090)* |  | -0.549 | 1.000 | 0.076 |
| **within**  ***(****0.078±0.059)* | **-** | **between**  ***(****0.122±0.080)* |  | -7.510 | < .001 | 4.105e+10 |
| **E1, within**  ***(****0.098±0.107)* | **-** | **E1, between****  *(0.142±0.151)* |  | -3.856 | 0.004 | 48.924 |
| **E2, within**  ***(****0.127±0.107)* | **-** | **E2, between****  ***(****0.131±0.141)* |  | -0.358 | 1.000 | 0.093 |
| **I1, within**  ***(****0.034±0.100)* | **-** | **I1, between****  ***(****0.111±0.119)* |  | -6.775 | < .001 | 1.264e+8 |
| **I2, within**  ***(****0.053±0.114)* | **-** | **I2, between****  ***(****0.103±0.114)* |  | -4.423 | < .001 | 1449.261 |
| *^ᵃ^ Greenhouse-Geisser correction was applied due to violated assumption of sphericity (p < .05)*  *Note.* Frequentist p-value adjusted for comparing a family of 6 (*) or 28 (**)  Underlined comparisons signal a change in significance with respect to the analysis with all participants | | | | | | |

Table **S11**. 4x2 repeated measures ANOVA (frequentist and Bayesian) with factors task and switch type performed on switch costs (with subtraction of task repetitions), to aid previous analyses in understanding the role of specific tasks in shaping the costs of switching between and within domains. 37 outlier participants excluded.

|  |  | **frequentist** | | | | **Bayesian** |
| --- | --- | --- | --- | --- | --- | --- |
|  |  | **t** | **df** | **p** | **Cohen’s d** | **BF_10_** |
| *A* | **within internal** | 7.905 | 186 | < .001 | 0.578 | 2.862e+10 |
|  | **between internal** | 16.811 |  | < .001 | 1.229 | 6.859e+35 |
|  | **within external** | 19.353 |  | < .001 | 1.415 | 1.034e+43 |
|  | **between external** | 16.598 |  | < .001 | 1.214 | 1.676e+35 |
| *B* | **E1, within** | 11.729 | 162 | < .001 | 0.919 | 1.901e+20 |
|  | **E1, between** | 11.969 |  | < .001 | 0.938 | 8.604e+20 |
|  | **E2, within** | 15.098 |  | < .001 | 1.183 | 2.897e+29 |
|  | **E2, between** | 11.886 |  | < .001 | 0.931 | 5.099e+20 |
|  | **I1, within** | 4.342 |  | < .001 | 0.340 | 564.543 |
|  | **I1, between** | 11.929 |  | < .001 | 0.934 | 6.685e+20 |
|  | **I2, within** | 5.914 |  | < .001 | 0.463 | 513324.314 |
|  | **I2, between** | 11.567 |  | < .001 | 0.906 | 6.904e+19 |

Table **S12**. One sample t-tests over switch types after subtracting repetitions to check that all switch types in the 2x2 (A) and 4x2 (B) rANOVAs were significantly different from task repetitions (=0). 13 (A) and 37 (B) outlier participants were excluded from the two analyses, respectively.

*8.2.3 Difficulty of task repetitions*

|  | | | **frequentist** | | | **Bayesian** |
| --- | --- | --- | --- | --- | --- | --- |
|  |  | **df** | **F** | **p** | **η² _p_** | **BF_10_** |
| **task repetition^a^** |  | 2.147, 403.568 | 29.502 | < .001 | 0.136 | 5.015e+14 |
|  | | |  | **t** | **p_bonf_** | **BF_10_** |
| **E1/consonants**  *(1.139±0.190)* | **-** | **E2/vowels**  *(1.055±0.170)* |  | 8.259 | < .001 | 3.970e+16 |
|  |  | **I1/personality**  *(1.090±0.174)* |  | 4.867 | < .001 | 257.241 |
|  |  | **I2/sensations**  *(1.057±0.169)* |  | 7.988 | < .001 | 4.127e+7 |
| **E2/vowels**  *(11.055±0.170)* | **-** | **I1/personality**  *(1.090±0.174)* |  | -3.391 | 0 .001 | 17.161 |
|  |  | **I2/sensations**  *(1.048±0.157)* |  | -0.271 | 1.000 | 0.084 |
| **I1/personality**  *(1.090±0.174)* | **-** | **I2/sensations**  *(1.057±0.169)* |  | 3.121 | 0.011 | 645.084 |
| *^ᵃ^ Greenhouse-Geisser correction was applied due to violated assumption of sphericity (p < .05)*  *Note.* Frequentist p-value adjusted for comparing a family of 6 (*)  Underlined comparisons signal a change in significance with respect to the analysis with all participants | | | | | | |

Table **S13**. Main analysis and post-hoc t-tests from the 1x4 repeated measures ANOVA (frequentist and Bayesian) with factor task repetition. This was used to check whether there was an intrinsic difference in task difficulty, irrespective of switch costs. 11 outlier participants were removed from this analysis.

*8.2.4 Switch costs grouped by difficulty*

|  | | **frequentist** | | | **Bayesian** |
| --- | --- | --- | --- | --- | --- |
|  | **df** | **F** | **p** | **η² _p_** | **BF_10_** |
| **Switch difficulty^a^** | 1.857, 358.361 | 2.528 | 0.085 | 0.013 | 0.212 |
| *^ᵃ^ Greenhouse-Geisser correction was applied due to violated assumption of sphericity (p < .05)*  *M±SD:  hard-to-easy = 0.106±0.089;  neutral = 0.120±0.095;  easy-to-hard = 0.102±0.098* | | | | | |

Table **S14**. 1x3 repeated measures ANOVA (frequentist and Bayesian) on switch costs with factor switch difficulty. This was used to check whether switch costs were influenced by task difficulty, irrespective of domain. 6 outlier participants were removed from this analysis.

*8.2.5 Effects of CTI duration*

|  | **frequentist** | | | | | **Bayesian** |
| --- | --- | --- | --- | --- | --- | --- |
|  |  | **df** | **F** | **p** | **η² _p_** | **BF_10_** |
| **domain** |  | 1 | 35.609 | < .001 | 0.167 | 9.679e+9 |
| **CTI** |  | 2 | 56.347 | < .001 | 0.240 | 4.165e+18 |
| **domain ✻ CTI** |  | 2 | 0.674 | 0.510 | 0.004 | 0.033 (BF_01_=30.537) |
|  | | |  | **t** | **p_bonf_** | **BF_10_** |
| **internal**  ***(****0.085±0.074)* | **-** | **external**  ***(****0.127±0.080)* |  | -5.967 | < .001 | 3.799e+10 |
| **short**  ***(****0.145±0.092)* | **-** | **medium***  ***(****0.092±0.072)* |  | 8.066 | < .001 | 6.202e+12 |
|  |  | **long***  ***(****0.079±0.074)* |  | 10.010 | < .001 | 6.319e+19 |
| **medium**  ***(****0.113±0.080)* | **-** | **long***  ***(****0.136±0.112)* |  | 1.945 | 0.158 | 0.624 |
| **int. short**  ***(****0.120±0.114)* | **-** | **int. medium****  ***(****0.073±0.093)* |  | 5.490 | < .001 | 10842.976 |
|  |  | **int. long****  ***(****0.060±0.098)* |  | 6.932 | < .001 | 6.367e+7 |
| **int. medium** ***(****0.073±0.093)* | **-** | **int. long****  ***(****0.060±0.098)* |  | 1.442 | 1.000 | 0.228 |
| **ext. short**  ***(****0.170±0.117)* | **-** | **ext. medium***  ***(****0.112±0.095)* |  | 6.762 | < .001 | 1.745e+8 |
|  |  | **ext. long****  ***(****0.099±0.095)* |  | 8.274 | < .001 | 9.506e+10 |
| **ext. medium** ***(****0.112±0.095)* | **-** | **ext. long**** ***(****0.099±0.095)* |  | 1.512 | 1.000 | 0.325 |
| *Note.* Frequentist p-value adjusted for comparing a family of 3 (*)  *Note.* Frequentist p-value adjusted for comparing a family of 15 (**)  Underlined comparisons signal a change in significance with respect to the analysis with all participants | | | | | | |

Table S15. 2x3 repeated measures ANOVA (frequentist and Bayesian) on switch costs with factors domain and CTI, to assess the influence of CTI duration on the magnitude of switch costs. 21 outlier participants were excluded.

|  | **frequentist** | | | | **Bayesian** |  |
| --- | --- | --- | --- | --- | --- | --- |
|  | **t** | **df** | **p** | **Cohen’s d** | **BF_10_** |  |
| **internal short** | 14.141 | 178 | < .001 | 1.057 | 4.895e+27 | |
| **internal medium** | 10.510 |  | < .001 | 0.786 | 2.218e+17 | |
| **internal long** | 8.277 |  | < .001 | 0.619 | 2.172e+11 | |
| **external short** | 19.410 |  | < .001 | 1.451 | 2.492e+42 | |
| **external medium** | 15.656 |  | < .001 | 1.170 | 1.026e+32 | |
| **external long** | 13.862 |  | < .001 | 1.036 | 7.750e+26 | |

Table S16. One sample t-tests over all conditions of the 2x3 rANOVA (domain x CTI), to check that all the switch costs were significantly different from task repetitions (=0). 21 outlier participants were excluded from the analysis.
