# Supplementary material for: Investigating the shift between externally and internally oriented cognition: a novel task-switching paradigm
Source: Neurosci Conscious. 2022 Nov 19;2022(1):niac016. doi: 10.1093/nc/niac016 (PMC9675616; doi:10.1093/nc/niac016)
Supplement: niac016_Supp [file niac016_supp.zip › OPEN SCIENCE BADGE APPLICATION FORM_Calzolari_Boneva_Fernandez-Espejo.docx]

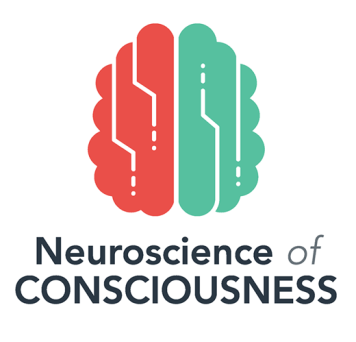
 **OPEN SCIENCE BADGE APPLICATION FORM**

**Open Data Badge**

Please provide the URL, DOI, or other permanent path for accessing the data in a public, open access repository.

I

Is there sufficient information for an independent researcher to reproduce the reported results? If no, explain.

**Open Materials Badge**

Please provide the URL, DOI, or other permanent path for accessing the materials in a public, open access repository.

I

Is there sufficient information for an independent researcher to reproduce the reported methodology? If no, explain.

**Preregistered Badge**

Please provide the URL, DOI, or other permanent path to the registration (and, if applicable, the analysis plan) in a public, open access repository.

I

https://osf.io/p7xrq

Was the plan preregistered prior to the examination of the data or observing the outcomes? If no, explain.

Yes, the plan was preregistered prior to data collection

Were there additional registrations for the study other than the one reported? If yes, provide links and explain.

No

For Preregistered and Analysis plan badge: were there any changes to the preregistered analysis plan for the primary confirmatory analysis? If yes, explain.

No

For Preregistered and Analysis plan badge: are all of the analyses described in the registered plan reported in the article? If no, explain.

Yes, all preregistered analyses are reported in the article. However, we also performed additional analyses beyond the ones we preregistered. This is all clearly stated in the manuscript, where we specifically label preregistered and non-preregistered analyses.
